# The Effect of a Structured Gastroenteritis Discharge Management Plan on Compliance, Prognosis, and Parents' Satisfaction

**DOI:** 10.7759/cureus.23240

**Published:** 2022-03-16

**Authors:** Mohammed Abuaish, Ghufran Mirza, Wijdan Al-Zamzami, Meshal Atiyah

**Affiliations:** 1 Department of Pediatrics, Faculty of Medicine, Umm Al-Qura University, Makkah, SAU; 2 Department of Pediatrics, Maternity and Children’s Hospital, Medina, SAU; 3 Department of Pediatrics, Johns Hopkins Aramco Healthcare, Dhahran, SAU; 4 Department of Pediatric Emergency, Maternity and Children Hospital, Makkah, SAU

**Keywords:** pediatric emergency department, parents' satisfaction, compliance, discharge instructions, gastroenteritis

## Abstract

Background: Acute gastroenteritis is one of the most common causes of dehydration in children. Parents’ education is an essential part of its management. In this study, we assessed the efficacy of discharge instructions in the pediatric emergency department for parents of children with acute gastroenteritis, together with disease prognosis and parents’ satisfaction.

Methods: An observational prospective cohort study was conducted among parents of children with acute gastroenteritis, with mild-to-moderate dehydration, who presented to the pediatric emergency room from March 2018 to July 2018. Parents were interviewed upon their child’s presentation and in follow-up phone calls after one week to assess the parents’ knowledge and the disease’s prognosis.

Results: There were a total of 218 parents of children with acute gastroenteritis of mild and moderate dehydration. The mean age was four years and one month ± three years and seven months. Forty-four percent of study participants had reasonable awareness of their child’s condition, and most patients (86%) improved fully. The exact adherence to instructions was 54%, the proportion of children who returned to the emergency department was 13%, and parental satisfaction and appreciation of the provided education was 98%.

Conclusion: In the study group, not strictly following fluid rehydration plans in discharge instructions did not negatively affect the course of improvement. This indicates that simple instructions to rehydrate with any fluid a child might accept and give clear red flags for observation are likely to be enough to treat gastroenteritis of mild-to-moderate severity.

## Introduction

Acute gastroenteritis is responsible for millions of deaths in young children each year, mostly in developing countries. It is a common presentation in both general practice and emergency departments and hospitals [1]. Dehydration may be associated with electrolyte disturbances and metabolic acidosis and is considered the most dangerous complication [1]. Optimal management with oral or intravenous hydration minimizes the risk of dehydration and its adverse consequences. However, routine use of antibiotics, antidiarrheals, and antiemetics is not recommended and can cause harm [1]. Prevention is vital in controlling gastroenteritis; the highly effective rotavirus vaccine has had a significant impact on public health [1]. Evaluation of acute gastroenteritis in children should include a recent history of fluid intake and output. Significant dehydration is unlikely if the parent reports no decrease in the oral intake or urine output and no vomiting or diarrhea. Physical examination is the best way to assess hydration status. The four-item clinical dehydration scale can be used to determine the severity of dehydration based on physical examination [2]. Microbiological stool tests are not routinely required for children with mild illness if viral gastroenteritis is the likely diagnosis, and mild gastroenteritis in children can be treated at home. Oral rehydration therapy is the main treatment for mild and moderate dehydration. If needed, ondansetron may be prescribed to prevent vomiting and improve the child’s tolerance of oral rehydration solutions. Hospitalization and intravenous fluids are recommended for children who do not respond to oral rehydration therapy or antiemetics, as well as for patients with severe dehydration [3]. Hand washing, breastfeeding, and rotavirus vaccination reduce the incidence of acute gastroenteritis in young children [3]. Additionally, educating parents influences the management of their children’s illnesses and increases their understanding of the disease, helping to avoid or minimize complications and reducing the frequency and duration of hospitalizations. Despite the importance of discharge communications in pediatric emergencies, the current practice is widely variable [4]. Many factors appear to affect the quality of the discharge communication and its effect with some conflicting results regarding the standardization of discharge communications in pediatric emergency departments [4,5]. Education can also improve a child’s quality of life and offers the significant potential benefit of reducing health care costs [6]. This outcome can be achieved through education by the discharging physician and properly supervised medical students. This also improves the likelihood of the parents adhering to discharge instructions, as proven in recent literature [7,8]. In this study, we aimed to determine the efficacy of a structured emergency department gastroenteritis management plan on compliance, prognosis, and parents’ satisfaction at a large pediatric tertiary care center in Makkah, Saudi Arabia. We assumed a positive relationship between medical student education and both adherence and parents' satisfaction.

## Materials and methods

An observational prospective cohort design was conducted in the pediatric emergency department (ED) of the Maternity and Children Hospital (MCH), Makkah, Saudi Arabia, from March 2018 to July 2018. The usual flow for cases of gastroenteritis in our ED starts in the triage area, where children come with symptoms and signs consistent with the preliminary diagnosis of gastroenteritis, such as vomiting, diarrhea, and absence of atypical features. The triage physician and nurse will supply an oral rehydration solution (ORS) to the child’s family to try to give to the child every five minutes in small volumes while waiting for the ED physician to assess the child. If the child fails to take the oral rehydration solution application, the triage doctor will give the child a dose of ondansetron and the family will be instructed to wait for 30 to 40 minutes before attempting oral hydration again. After the child is assessed and examined by the ED physician and the diagnosis of gastroenteritis with mild-to-moderate dehydration is confirmed using the clinical dehydration scale (CDS), the parents are usually educated by the ED physician before the child is sent home [2]. In our study, the clinic next to the ED physician’s office and assessment zone was used for the research team to conduct the education process. After the ED physician discharged the patient, the research team approached the parents, and if they agreed to be enrolled in the study, they moved them to the education office. At the office, they spent around 15 minutes going over the detailed printed structured discharge form, which educates the family on how to use the ORS and the volume to be given in the next 24 hours (maintenance fluid) and how to replace ongoing loses of vomiting and diarrhea with ORS, in addition to the red flags that the family should watch for to come back to the ED. All patients were given the same type of oral rehydration solution.

The study was conducted through interviews with parents. Institutional review board approval was obtained from Umm Al-Qura University’s Biomedical Ethics Committee (approval number: HAPO-02-K-012-2020-07-423). Medical students and authorship members who collected the data underwent multiple training sessions before administering the survey to ensure that they were competent and sufficiently educated to handle parents’ questions and guide the discussion. The data collection tool was developed and designed by the research authors and built with the guidance of other studies [7-9]. It was reviewed and edited by a pediatric emergency physician, a clinical statistician, and a medical educator. Finally, it was examined and modified after the pilot study.

Inclusion and exclusion criteria

Children under 14 years of age who visited the ED and were diagnosed with acute gastroenteritis (per ED physician) by the presence of a constellation of symptoms and signs of acute gastroenteritis, had mild-to-moderate dehydration as per the clinical dehydration scale (CDS), and whose parents were willing to participate were included in this study [2]. In contrast, we excluded children who had severe dehydration, gastroenteritis with comorbidity, or were presented after their condition was seen and managed at other healthcare facilities. A physician conducted the initial assessment and confirmation of the diagnosis of acute gastroenteritis, and parental consent was obtained.

Study participants

The study participants were parents of a child with acute gastroenteritis who presented with mild-to-moderate dehydration. The two interviews were conducted with the same parent to minimize the bias of multiple data sources. In a separate clinic, the parents of the patients who fulfilled the inclusion criteria were interviewed and counseled regarding their children's condition and educated about acute gastroenteritis (AGE), its symptoms, the oral rehydration therapy treatment, and how to use it. The mother or father attended the education session and the same parent (the main caregiver who is presenting with the child majority of the time) provided their number for the follow-up phone interview. The parent’s questions were answered using the teach-back technique, and the parents were given the printed detailed structured strict rehydration plan (Appendices). After one week, the parent (the main caregiver) was contacted by phone to participate in a phone interview using another data collection form for follow-up information. The data collection form consisted of a coding system table that included patients’ initials and codes, which aided in referring to patients during the interview and the follow-up. The first part of the form collected demographic data, including age and gender. The latter part included questions about the patient's status, vomiting, further visits to the ED, and responses to treatment, as well as compliance with ORS, the type of ORS used, and parental satisfaction with disease awareness. Regarding data analysis and sample size estimation, it was a convenient sample of all the patients who presented with gastroenteritis from March 2018 to July 2018. Data were expressed as mean ± standard deviation and analyzed by IBM SPSS Statistics for Windows version 23 (Armonk, NY: IBM Corp.). Pearson’s chi-squared test and Fisher’s exact test were used to calculate the associations between groups. P-values of <0.05 were considered statistically significant.

## Results

The study included 218 children who fulfilled the inclusion criteria, 115 males (52.8%) and 103 females (47.2%). Parents of 218 children were interviewed and educated. Around 44% of parent participants were aware of their child’s correct diagnosis, while the rest thought that other illnesses could have caused their child’s presentation. From the overall questions that assessed knowledge about the disease, around 25% of participants were aware of the disease and had a good background on what could cause it and how it should be treated (Table 1). The second interview was conducted one week later. The main theme of the second interview focused on the child’s improvement and the parents’ experience and compliance with the proposed treatment (Table 2).

**Table 1:** Characteristics of patients and awareness. ORS: oral rehydration solution

Characteristics	Value
Patients’ age (years)	4.09±3.61 (0.25-15.00)
Patients’ gender	
Male	115 (52.8%)
Female	103 (47.2%)
Q1. What do you think your child has?	
Gastroenteritis	96 (44.0%)
I don’t know	44 (20.2%)
Cold/flu	21 (9.6%)
Food poisoning	19 (8.7%)
Pharyngitis	15 (6.9%)
Teething	12 (5.5%)
Other	10 (4.6%)
Pneumonia	1 (0.5%)
Q2. From the beginning of your child’s symptoms, how many ER/doctor visits have you made (including this visit)?	
First visit	167 (76.6%)
Second visit	42 (19.3%)
Third visit	5 (2.3%)
Fourth visit	4 (1.8%)
Q4. Have you used ORS before coming to the ER?	
No	195 (89.4%)
Yes	23 (10.6%)

**Table 2:** The effect of education at the clinic on parents’ compliance and subsequent health recovery of the patient. ORS: oral rehydration solution

Questions	N (%)
Q1. Fluids received?	
ORS	185 (84.9%)
Water	60 (27.5%)
Milk	46 (21.1%)
Juice, soup, others	67 (30.7%)
Q2. How was ORS given?	
According to the dosage and manner explained by the doctor	119 (54.6%)
When requested or when accepted	61 (28.0%)
Did not drink the rehydration solution	36 (16.5%)
Others	2 (0.9%)
Compliance	119 (54.6%)
Non-compliance	99 (45.4%)
Q3. Which type of ORS did your child accept?	
Pre-made ORS only	121 (55.5%)
Powder ORS only	45 (20.6%)
None (did not accept or use ORS)	26 (11.9%)
Both Pedialyte and powder	3 (1.4%)
Other	23 (10.6%)

**Figure 1:**
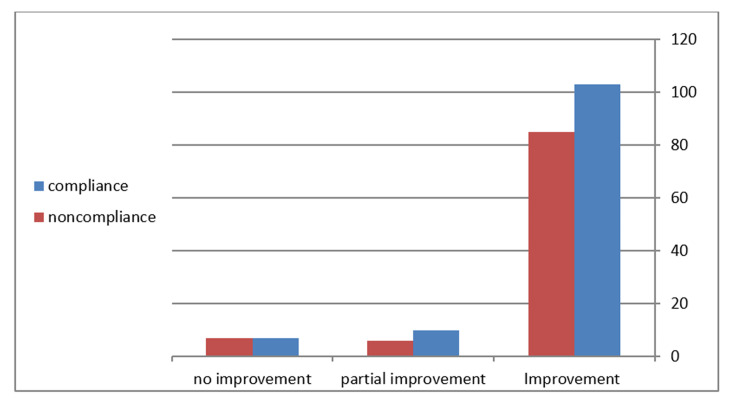
Compliance and improvement of children with acute gastroenteritis.

Regarding patient improvement, around 86% (n=188) of the patients improved completely according to parents’ response, 7% (n=16) mentioned that they had only attained partial improvement, and 6% mentioned that their symptoms had not yet resolved. In 0.5% (n=1) of the cases, the patient was readmitted to the hospital. A return to the emergency department was observed in 13.8% (n=30) of the study sample. The main reason for returns to the ED was vomiting (76% {n=23 of 30 visits}), while other causes were less frequently observed. The main fluid used at home for rehydration management was ORS, in 84.9% (n=185) of cases, while other types of fluid used included water, juice, milk, and soup. Regarding parents’ compliance with the education plan, 54% (n=119) mentioned that it was given as instructed upon discharge and that they had fully adhered to the discharge plan, while 28% (n=61) gave ORS if the child asked for it or when their child accepted it. Sixteen percent (n=36) of the study population did not give the oral rehydration solution at all (Figure 1). All groups were similar regarding recovery and return to ED, with the majority improving in the first 24 hours. The majority of the participating parents, 98.6% (n=215), were happy with the manner of teaching and education conducted in the emergency department and stated that they wanted this education to continue with any potential future emergency visit (Tables 2, 3).

**Table 3:** Parents’ experience of the emergency visits and the management course. ED: emergency department

Questions	N (%)
Q1. Presence of vomiting at the presentation?	
Yes	57 (26.1%)
No	161 (73.9%)
Total frequency of vomiting during the illness	
1	19 (8.7%)
2	17 (7.8%)
3 or more	21 (9.6%)
Q2. Return to ED or other hospitals the next day?	
No	188 (86.2%)
Yes	30 (13.8%)
Numbers of return visits	
1	28 (12.8%)
2 or more	2 (1%)
Q3. Had they received education before?	
Yes	41 (18.8%)
No	175 (80.3%)
I don't remember	2 (0.9%)
Q4. Did they prefer to continue education?	
Yes	215 (98.6%)
Maybe	3 (1.4%)
No	0 (0%)

## Discussion

Gastroenteritis is a relatively common presentation to the pediatric emergency department, which nevertheless carries significant anxiety for parents [10]. In this study, we considered the efficacy of our education of parents and its effect on children’s health, the use of oral rehydration solution, and the way that parents used it.

How effective were the recommendations that we gave to the parents? In our study, it was clear that parents needed this information because less than half of the parents recognized that the symptoms were caused by gastroenteritis; this fact is noted in several other studies as well [11]. Despite poor adherence of the caregiver to the detailed structured discharge planning and strict ORS use instructions, the majority of children did well and recovered in the next 24-48 hours from their illness. This supports the known evidence that strict discharge instructions for an oral rehydration solution are not always recommended, as long as the child can take variable other oral solutions [12,13] (Figure 1). Part of this could be because of the viral nature of the illness, that it included patients who were experiencing acute gastroenteritis with mild-to-moderate dehydration, and that the expected outcome was that around 90% of the symptoms would improve [14]. In our study, 86% improved completely. However, the complexity of the instructions given and the difficulty associated with delivering this information to the parents of a sick child makes it very difficult for parents to adhere to the instructions. This problem has been noted before in several studies of pediatric emergency care [12,15].

In 2011, Freedman et al. conducted a prospective, single-blind trial to determine the effectiveness of intensive gastroenteritis education programs on caregivers of children with gastroenteritis. They concluded that an intensive ED and home gastroenteritis education program did not result in a long-term increase in the disease-specific caregiver knowledge [16]. As in our study, the education did not affect the disease prognosis nor the adherence of parents to the given plan.

In 2009, Patel et al. conducted a study to assess the use of a discharge facilitator in improving recall of emergency department discharge instructions for acute gastroenteritis and concluded that it would improve parental recall of discharge instructions for gastroenteritis and increase their satisfaction in comparison to standard discharge protocol [17]. As observed in our study, satisfaction was high among parents who underwent education sessions. However, we did not have a control group to compare to. In the current literature, there is a lack of consensus on the way to ensure parent comprehension and adherence to discharge instructions [18]. And that is a great challenge in creating a satisfying way to build a standardized discharge instruction plan that is convenient to the healthcare provider and the caregivers [19]. In our study, the detailed discharge instructions were well accepted by parents but full adherence was not observed. Parents were happy and satisfied with the given education. However, as observed in other studies, that was not enough for them to adhere completely to the given plan [17,19]. In our study, the parents’ degree of satisfaction probably indicates the appetite for health education received from multiple sources, including the media and school, not just from the emergency department. And this might lead us to another question regarding the factors that affect the parents’ adherence to the discharge instructions and their relation to plan complexity and health literacy, as noted by Glick et al. in 2019 [15].

One of the main challenges to providing a well-tolerated discharge plan for gastroenteritis is the parents’ attitude toward oral rehydration therapy and whether they accept it as a way of therapy or prefer other methods. This was observed by Nir et al. in 2013, who conducted a study to evaluate parents’ attitude toward oral rehydration therapy and found that half of their study population refused the oral rehydration, as they were expecting other procedures [20]. In our study, the oral rehydration solution was accepted initially, but not followed strictly as planned, especially if an improvement was already observed. Medical students are an important part of the health care system and clinical training is an integral part of their education. It cannot be substituted with other educational activities [9,21,22]. Involving medical students in daily clinical practice is a useful way to enhance their knowledge and widen their exposure if it is done well and with proper supervision. In addition, medical student involvement in patient education has significant benefits because the students will spend more time engaged in clinical practice and answering parents’ questions. In previous studies, education by students has been associated with clinical and educational benefits in comparison to patient education by emergency physicians [7,21,23]. In our study, however, no comparison was made, and further studies are needed both to evaluate the exact role of medical students in patient education and to identify further gaps in patient communications in the pediatric emergency department.

Study limitations

One of the main limitations of this study is that it was conducted with cases of mild-to-moderate gastroenteritis. Including severe gastroenteritis might add to the evaluation of the efficacy of the parents’ education, but it would also require a follow-up for the hospital course during the admission period, which would have been beyond the scope of the study and what it was intended to measure. Another limitation is that it used a data collection form that depends on participants’ memory and impressions, which has less accuracy in comparison to more objective data and might lead to more reporter bias. However, this method was needed to obtain the parents’ impressions and feelings toward the education process. Another limitation is that there was only one parent of each patient who participated in the study who was the main caregiver for the patient. Including both parents of all patients in a detailed manner will add more value to the data and the concluded results. In our study, this did not apply to all patients because the main caregiver was usually the parent who presented themselves with the patient at the ED visit. The last limitation is that we should take the recommendation of using any fluids compared to oral rehydration solutions carefully because our study population consisted mostly of patients with mild gastroenteritis who presented with less electrolyte disturbance than in cases of severe gastroenteritis. Therefore, the generalization of our results should not be applied to those cases of severe gastroenteritis that may need more electrolyte-rich oral rehydration solutions.

## Conclusions

A strict fluid rehydration plan with WHO oral rehydration solutions in the discharge instruction is not necessary for patients with mild-to-moderate gastroenteritis neither they did affect the outcome of those patients. Costly oral rehydration solutions did not seem to affect the favorable outcome of those patients who presented with mild-to-moderate gastroenteritis. What seems to be more important is simple instruction plan of using any oral fluids the child tolerates and accept to prevent dehydration in the next 24-48 hours after the discharge of the emergency department for kids with mild-to-moderate gastroenteritis as well as the importance of following up the signs of dehydration, oral intake and urine output as more objective signs of dehydration. Discharge recommendations in the acute setting can be provided by medical students under proper supervision and can be established in the acute setting of the pediatric emergency department. Further studies are needed to compare the efficacy of education provided by medical students with the education provided by medical educators or emergency physicians in the same setting.

## Appendices

**Table 4:** Educational material that was used for parents during this study.

If your child is older than six months and he has no signs of dehydration you can take care of him at home. The rehydration solution should be prepared in 1 liter of water or juice and should be given according to your child's weight as follow:	Remember: If your child is having fever he should receive antipyretic every four to six hours and if his temperature is more than 38° C and sponges as directed by your doctor. Your child may get diaper rash secondary to his diarrhea, you may need to change his diaper more frequently than you usually do to avoid the rashes. Remember to wash your hands and your child's hands and avoid contact with sick children to decrease infection transmission. You should consult your doctor immediately if one of the following happens: (i) If diarrhea continued for more than 10 days. (ii) If the vomit or the loose stool has blood. (iii) If the abdominal pain is severe. (iv) If your child got a fever up to 40° C. (v) If your child's activity decreases.
Your child's weight is …….. kg. He/she should take …..... ml during the day, divided into small amounts. And he/she should receive additional …… ml with every loose stool or vomit he gets.	
The recommended solutions for your baby's hydration are as follows: oral rehydration solutions, water, juices that are prepared at home to avoid having more diarrhea from high sugar content juices.	
The recommended meals are as follows: yogurt, mashed potato, carrots, apple, fruits, rice, boiled chicken, and bread (if his age allows). Things to avoid: candies, full-fat milk, fizzy drinks, french fries, and ice-creams. Don't give any anti-diarrhea medication.	
